# Morphogenetic Mechanisms in the Cyclic Regeneration of Hair Follicles and Deer Antlers from Stem Cells

**DOI:** 10.1155/2013/643601

**Published:** 2013-12-07

**Authors:** Chunyi Li, Allan Pearson, Chris McMahon

**Affiliations:** ^1^AgResearch Invermay Agricultural Centre, Private Bag 50034, Mosgiel 9053, New Zealand; ^2^State Key Laboratory for Molecular Biology of Special Economic Animals, Chinese Academy of Agricultural Sciences, Changchun, Jilin 130112, China; ^3^AgResearch Ruakura Agricultural Centre, Private Bag 3123, Hamilton 3240, New Zealand

## Abstract

We have made comparisons between hair follicles (HFs) and antler units (AUs)—two seemingly unrelated mammalian organs. HFs are tiny and concealed within skin, whereas AUs are gigantic and grown externally for visual display. However, these two organs share some striking similarities. Both consist of permanent and cyclic/temporary components and undergo stem-cell-based organogenesis and cyclic regeneration. Stem cells of both organs reside in the permanent part and the growth centres are located in the temporary part of each respective organ. Organogenesis and regeneration of both organs depend on epithelial-mesenchymal interactions. Establishment of these interactions requires stem cells and reactive/niche cells (dermal papilla cells for HFs and epidermal cells for AUs) to be juxtaposed, which is achieved through destruction of the cyclic part to bring the reactive cells into close proximity to the respective stem cell niche. Developments of HFs and AUs are regulated by similar endocrine (particularly testosterone) and paracrine (particularly IGF1) factors. Interestingly, these two organs come to interplay during antlerogenesis. In conclusion, we believe that investigators from the fields of both HF and AU biology could greatly benefit from a comprehensive comparison between these two organs.

## 1. Introduction

Hair follicles (HFs) and deer antlers are the only two mammalian organs capable of stem-cell-mediated cyclic regeneration in adult life [1, 2]. After a careful examination of the literature, we have found that these two organs share some interesting commonalities. Moreover, an interplay between these two organs is required for the development of antlers (antlerogenesis). This review briefly describes the processes of organogenesis and cyclic regeneration of HFs and antlers, identifies their similarities and differences, reveals intercommunication between the two organs during antlerogenesis, and presents some points of common interest in which the two research fields could mutually benefit.

**Figure 1:**
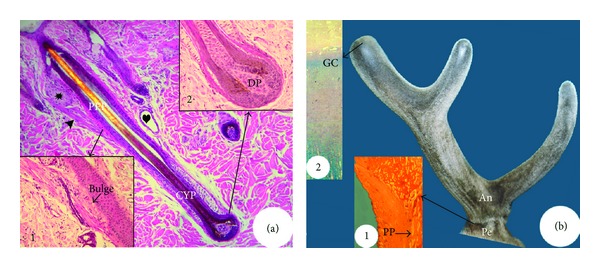
Structure of a mature hair follicle (HF) at the late anagen phase (a) and an antler unit (AU) at the growing phase (b). HF consists of a permanent part (PEP) and a cyclic part (CYP). The bulge ((a), Inset 1) locates at the site where arrector pili muscle (arrow head) attaches to the permanent part and contains HF stem cells, and the bulb ((a), Inset 2) at the proximal end of the cyclic part and contains the growth centre including dermal papilla (DP). HF also contains a sebaceous gland (asterisk) and a sweat gland (heart). AU consists of a permanent part (pedicle, Pe) and a cyclic part (antler, An). The pedicle periosteum (PP; (b), Inset 1) envelops pedicle bone and contains antler stem cells, and the growth centre (GC; (b), Inset 2) locates in the tip of a growing antler.

A typical mature HF (Figure 1(a)) can be divided into two parts: a permanent distal part (proximity to epidermis) and a cyclic proximal part (away from epidermis) [3]. The permanent part consists of the infundibulum and the isthmus. These two subparts are delineated at the junction with the sebaceous gland duct. An arrector pili muscle is attached to the outer root sheath of an HF at the proximal end of the isthmus, where a special structure called the bulge is located (Figure 1(a), Inset 1). The bulge harbours stem cells and marks the proximal end of the permanent part during regeneration of the HF [4]. The cyclic part includes the proximal shaft called the suprabulbar strand and the bulb (Figure 1(a), Inset 2), where the growth centre of the HF resides [5]. The bulb contains matrix keratinocytes, melanocytes (pigmentary units), and dermal papilla (DP) cells (the closely packed mesenchymal cells). The bulge (stem cell niche) and the bulb (growth centre) are separated by a long segment of suprabulbar epithelium. The HF shaft consists of multiple epithelium-derived layers arranged concentrically. Starting from the periphery, these layers are the outer root sheath (the basal layer of the follicle), the companion layer, the inner root sheath, and finally the hair fibre [6]. The entire epithelium of the hair follicle is surrounded by a mesoderm-derived connective tissue sheath [7], which is in continuity with the DP in the hair bulb (Figure 1(a)).

In this review, we define antler unit (AU) as a term for both antler proper and antler pedicle (Figure 1(b)), whereas the term “antler” denotes antler proper. The pedicle is the permanent part of the AU and remains as a bony stump following antler casting each year [8, 9]. The pedicle bone is ensheathed in a layer of periosteum (pedicle periosteum, PP), within which reside stem cells for regenerating the antler (Figure 1(b), Inset 1 [10]). The antler is the cyclic part of the AU and includes the main beam and a number of lateral projections called tines (the number and formation of which vary with age and among deer species). The growth centres of a growing antler are located in the tip of the main beam and in the tip of each tine (Figure 1(b), Inset 2 [11, 12]). AU consists of five concentric layers. Starting from the periphery, these layers are the epidermis, dermis, periosteum, cortex, and, finally, the medulla [11, 13]. Pedicles and antlers are delineated by the type of skin. Specifically, pedicles are enveloped by typical scalp skin, while antlers have a unique velvet-like skin that is sparsely populated with hair and is known as velvet (Figure 1(b)).

In summary, both HF and AU have permanent and cyclic components. The permanent component of each organ harbours its respective stem cells, and the cyclic component contains the growth centre for the formation/regeneration of each organ. The entire HF organ is ensheathed in a mesoderm-derived connective tissue, whereas the AU is in an epithelium-derived epidermis.

## 2. Ontogeny

The ontogeny of both HF and AU includes organogenesis and cyclic regeneration.

### 2.1. Organogenesis

#### 2.1.1. HF

**Figure 2:**
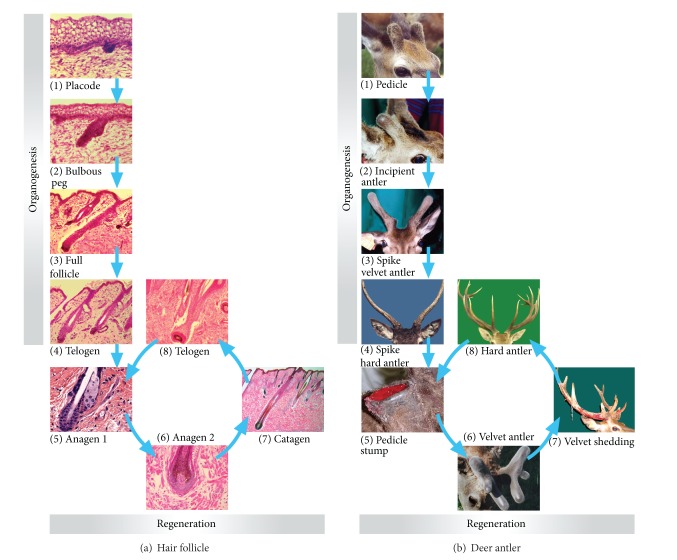
Ontogeny of HF and AU (for detailed descriptions, refer to the text). (a) Ontogeny of HF. (a)(1) Epithelial placode; (a)(2) bulbous peg; (a)(3) mature HF; (a)(4) HF at telogen; (a)(5) HF at anagen I; (a)(6) HF at anagen II; (a)(7) HF at early catagen; (a)(8) HF at telogen. (b) Ontogeny of AU. (b)(1) Fully grown pedicles; (b)(2) antler initiation from a fully grown pedicle; (b)(3) half-grown spike antlers; (b)(4) dead hard spike antlers; (b)(5) hard antler casting; (b)(6) antlers at mid regenerating stage; (b)(7) velvet skin shedding; (b)(8) hard regenerated antlers.

Based on morphological features, Paus et al. [5] classified organogenesis of the marine HF into eight stages. The initial stage is the development of an epithelial placode (Figure 2(a)(1)), a morphologically recognizable epidermal thickening. At stage 2, the hair germ develops into a more prominent and enlarged column of epidermal keratinocytes. This column has a convex proximal end, delineated by a discernable “cap” of mesenchymal cells. Stage 3 is characterised by the formation of a solid hair peg. The mesenchymal cells are now recognizable as a ball-shaped aggregation at the proximal end of the epithelial column termed the DP. At stage 4, the hair peg becomes elongated and acquires a bulb-like thickening at the proximal end within which the DP is situated in a prominent cavity formed by the surrounding hair matrix. At this stage, the pale epithelial layer of the inner root sheath starts to develop above the DP. During stage 5, also known as the bulbous peg stage (Figure 2(a)(2)), the inner root sheath elongates to reach half the length of the final hair follicle, and the site for the future stem-cell reservoir starts to enlarge into a bulge. The first sebocytes begin to appear above the bulge region, indicating that formation of the sebaceous gland has been initiated. The DP is now almost completely enclosed by the developing HF bulb. At stage 6, the hair canal becomes visible and the multilayered inner root sheath extends to the level of the hair canal that now contains a hair shaft with visible melanin granules in the proximal hair shaft. Stage 7 is characterised by the tip of the hair shaft leaving the inner root sheath and entering the hair canal at the level of the infundibulum of the enlarged sebaceous gland, which is now located on the posterior wall of the HF. At stage 8, the HF has acquired its maximal length and a prominent hair shaft emerges through the epidermis (Figure 2(a)(3)). The bulge acquires its distinctive appearance when the first postnatal hair germ emerges [14].

Renewal of the follicle and replacement of the pelage occur subsequently, at varying times depending on the follicle type and species, with the cyclic HF components rapidly degenerating via a process involving apoptosis. This stage of the follicle growth cycle is termed catagen. An epithelial strand surrounded by the retracting basement membrane draws the DP upward, where it comes to rest just below the bulge [1]. Upon completion of catagen, the HF enters a stage of relative quiescence known as telogen (Figure 2(a)(4)).

#### 2.1.2. AU

Pedicles develop from the presumptive region of the frontal bone (behind and above the eye) when male deer approach puberty [15, 16]. Initially, an incipient pedicle is covered by scalp skin (Figure 2(b)(1)). When the pedicle reaches a species-specific height (around 5 cm in red deer), the shiny velvet skin is formed on the apex (Figure 2(b)(2)). The change in skin type from scalp to velvet indicates the termination of pedicle formation and the initiation of antler growth. Rapid antler growth takes place in the late spring and early summer (Figure 2(b)(3)). In early autumn, antlers stop growing and become calcified, which triggers the shedding of velvet. In winter, the dead and bony antlers are firmly attached to their living pedicles (Figure 2(b)(4)).

During antler growth, the AU consists of an internal bony component and an external skin component. Formation of the internal component proceeds through four histologically distinguishable stages [9]. The first stage is called intramembranous ossification. At this stage, cells of the antlerogenic periosteum (AP) overlying the frontal crest (where pedicle growth is initiated) start to proliferate and differentiate into osteoblasts to form trabecular bone. When the pedicle exceeds 5 mm in height, some of the apical cellular layer cells of the AP begin to differentiate into chondroblasts. This stage is termed transitional ossification and osseocartilaginous tissue is formed. When the pedicle grows to 25–30 mm in height, almost all the cells of AP apical layer have differentiated into chondroblasts and cartilage tissue has formed. This stage is termed pedicle endochondral ossification. When the pedicle reaches the species-specific height (marked by the change in the external skin type) and transforms into an antler, cellular layer cells in the apical AP continue to differentiate into cartilage tissue until the entire antler is fully formed. This stage is called antler endochondral ossification. The pedicle and antler endochondral ossifications are histologically indistinguishable and both belong to a type of modified endochondral ossification because vascularised cartilage, rather than classical avascular cartilage, is formed.

Pedicle skin forms from the skin overlying the frontal crest and proceeds through three distinctive stages: (1) compression of the subcutaneous loose connective tissue at the transitional stage, (2) stretching of the undulated apical epidermis at the early pedicle endochondral ossification stage, and (3) neogenesis of the skin and the associated HFs at the mid pedicle endochondral ossification stage [17]. The transformation from pedicle to velvet skin occurs at the late pedicle endochondral ossification stage and is associated with changes in the types of HF. These changes include loss of arrector pili muscles and sweat glands and a gain of the large bi- or multilobed sebaceous glands.

In summary, both HF and AU undergo organogenesis to generate permanent and cyclic components. The permanent component of each organ is formed first and then the cyclic component is formed. It should be noted that in the HF, the distal part is permanent and the proximal part is cyclic, while the converse is true for the AU because the HF grows inwards and AU grows outwards. Organogenesis of both HF and the AU involves two principle types of cells: epithelium and mesenchyme. However, the HF is essentially an epithelial structure, while the AU is essentially a mesenchymal outgrowth. Each tissue is formed primarily from the cell type that is destined to constitute the final appendage, that is, hair in HF and antler in AU.

### 2.2. Regeneration

#### 2.2.1. HF

Each cycle of hair regeneration begins when proliferating hair germ cells emerge from the bulge at the end of telogen to commence the active growth phase (anagen). The shedding of the existing hair fibre (exogen), at or following anagen onset, was initially thought to be due to the outward movement of the nascent hair fibre [27], but it is now known that exogen in mouse HF involves activation of proteolytic processes [28]. The progression to form a mature HF in cyclic regeneration recapitulates ontogeny of the initial HF organogenesis [29].

At anagen, matrix cells, the transient amplifying cells, derived from HF stem cells of the bulge in human HF, proliferate at an astonishing rate (Figures 2(a)(5) and 2(a)(6)), having mitotic indices comparable to bone marrow and intestinal epithelium [30]. However, matrix keratinocytes stop proliferating after the new hair fibre is fully formed when the follicle enters the brief catagen transition phase (Figure 2(a)(7)), marked by extensive regression of the cyclic part of the HF and leading to quiescence (telogen) (Figure 2(a)(8)).

#### 2.2.2. AU

Each spring, hard antlers formed in the previous year are cast from their pedicles (Figure 2(b)(5)) in a process induced by the activation of osteoclasts in response to a reduction in the concentration of circulating androgens [31–33]. Wound healing takes place on the pedicle stumps immediately after casting, following which regeneration of antlers ensues in a process that recapitulates the development of the first antlers [34, 35] (Figure 2(b)). However, in subsequent cycles of antler regeneration, tines develop from the main beam to form a species-specific configuration.

In summary, at the catagen/telogen phase in the HF or the casting phase in the AU, the responsive cells (DP cells in HF or epidermal cells in AU) migrate to the proximity of the stem-cell niche (bulge in HF or PP in AU) to form a close association with their respective stem cells. Shedding of hairs or casting of hard antlers requires active proteolysis. Histologically, regeneration of each organ recapitulates the process of its respective organogenesis. Generally, loss of hair or hard antlers coincides with the onset of regeneration of each new organ.

## 3. Stem-Cell-Based Process

The cyclic components of HF and AU periodically regress and regenerate. For this to occur, there must be a population of stem cells residing in the permanent component of each organ. Furthermore, as the HF is principally an epithelial structure and AU is a mesenchymal structure, the tissue-specific stem cells required should be of the same lineage, that is, epithelial and mesenchymal, respectively. Tissue/cell deletion and transplantation experiments have played an important role in discovering and characterising the tissue-specific stem cells for both organs.

**Figure 3:**
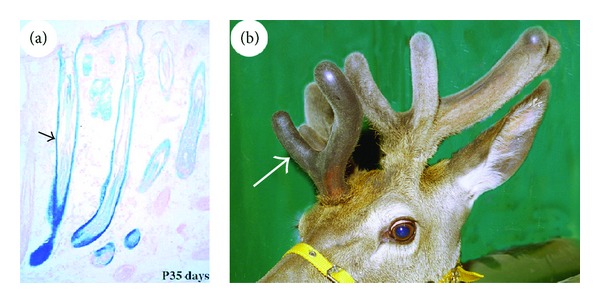
Stem-cell/tissue transplantation and ectopic organogenesis. (a) HF (arrow) was formed from the bulge that was transplanted inside the fetal dermal tissue (reproduced with permission from [18, Figure 4D]). (b) Antler (arrow) was formed from the antlerogenic periosteum (AP) that was subcutaneously transplanted onto the forehead of a male deer calf.

Studies that have either deleted or transplanted key components have played an important role in elucidating the tissue-specific stem cells for both HF and AU [4]. Oliver [36] reported that amputation of less than 1/3 length of the distal part (including bulb and the bulbar proximal part) of an HF (rat whisker) results in regeneration from the remaining distal component. Furthermore, deletion of the bulge region from the permanent component of an HF resulted in miniaturisation or aborted growth, whereas transplantation of the bulge tissue into foetal dermis causes formation of all the HF epithelial lineages [18] (Figure 3(a)). Even when cells of the bulge are transplanted to the dermis of foetal skin, ectopic mouse HFs can be induced to grow [37, 38]. These bulge cells of human HF express the key embryonic stem-cell markers: Oct4, Nanog, and SOX2 [39, 40] and the progeny of these stem cells contribute to all HF epithelial lineages. Recently, it has been reported [41] that mouse HF stem cells are specified even before bulge formation during HF morphogenesis (placode) and represent the direct precursors of the cells that reside in the mature bulge. HFs and sebaceous glands do not develop in the absence of these early HF stem cells.

In AU, when the periosteum overlying a frontal crest (AP) or enveloping a pedicle (PP) is removed, no AU is formed and antlers cannot regenerate. When the AP is transplanted elsewhere subcutaneously, an ectopic AU will form and subsequent antler regeneration will ensue [42–44] (Figure 3(b)). In experiments to date, it has not been possible to induce ectopic antler generation/regeneration by transplanting PP tissue [45]. However, when the PP was partially deleted, regeneration took place from the distal end of the PP even if it is located on the midsection of a bony pedicle shaft, that is, a site that is markedly distant to the original regeneration site (pedicle cast plane) [10]. Although no attempt has been made thus far to transplant singular AP cells, a mixture of finely minced AP (up to 200 *μ*m in thickness, unpublished) transplanted either subcutaneously or intradermally can initiate growth of an ectopic antler [46, 47]. Notably, the periosteal cells also express the key embryonic stem-cell markers Oct4, Nanog, and SOX2 [45, 48]. Therefore, AP cells are the stem-cell population required for organogenesis of AU, and PP cells are the stem cells required for regeneration of antlers.

**Figure 4:**
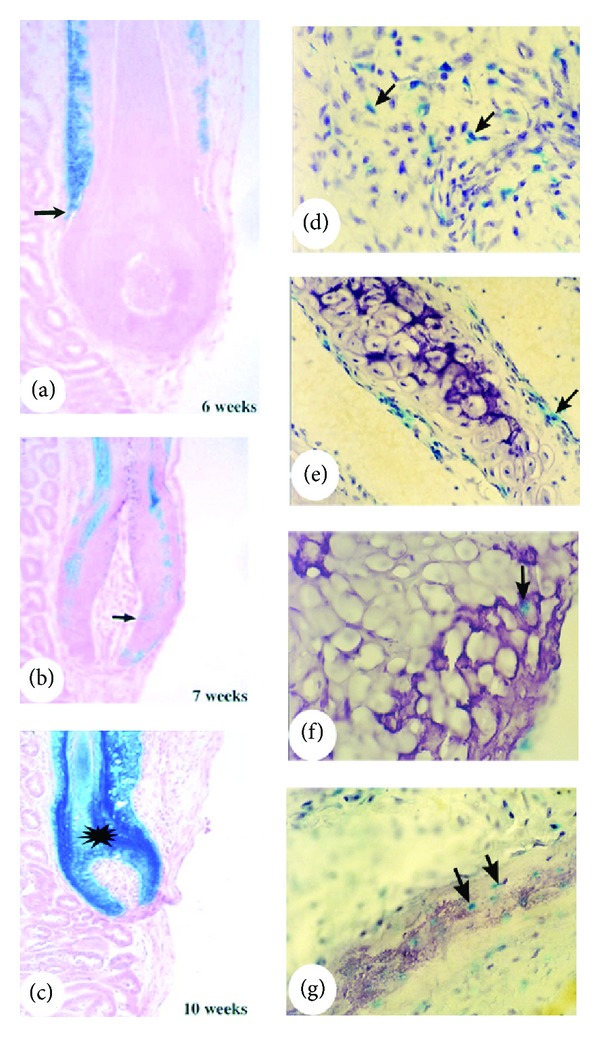
Stem-cell lineage tracing using an exogenous gene LacZ. (a)–(c) Reproduced with permission from [18, Figures 3A, 3D, and 3F, resp.]. Chimeric HF that was created by a wild type HF having its bulge being replaced with the one that expresses LacZ. Note that the *β*-galactosidase-positive cells gradually moved down the HF shaft, reaching the bulb region at the sixth week (a), the tip of the bulb at the seventh week (b), and the entire HF at the tenth week (c). (d)–(g) Reproduced with permission from [19, Figures 3F, 3G and 3H, resp.]. Histological sections from the four areas of a growing antler, which was formed from AP of the presumptive AU region where a small population of AP cells was labelled by LacZ gene. Note that *β*-galactosidase-positive cells were detected in every mesenchyme-derived tissue component of the antler (excluding the skin) including reserve mesenchyme (d), precartilage (e), cartilage (f), and lamellar bone (g).

Cell lineage tracing studies using the genetic marker gene LacZ (encoding *β*-galactosidase) have further confirmed that the stem cells required for development of the mouse HF are located in the bulge [18] and for development of the AU are located in the AP [19]. When the bulge tissue of an HF was replaced with the one that expresses exogenous gene LacZ and transplanted into nude mice, the *β*-galactosidase-positive cells gradually migrated down the HF shaft from the bulge and became juxtaposed to the proximal end of the follicle. Six weeks after transplantation, the *β*-galactosidase-positive cells had migrated to the bulb region (Figure 4(a)). At the seventh week, the cells had reached the tip of the bulb and commenced participation in the formation of the HF matrix (Figure 4(b)). At the tenth week, the cells had contributed to all the epithelial lineages involved in the formation of an HF (Figure 4(c)).

**Figure 5:**
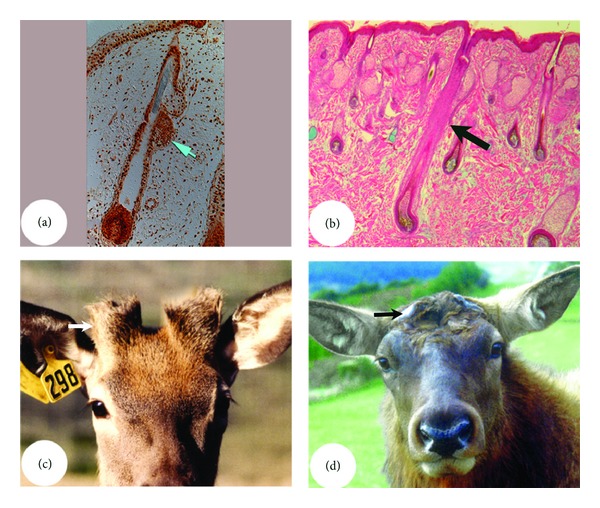
Influence of animal age on the size of the stem-cell niche. (a) and (b) HF bulges. Note that bulge is a very prominent structure (arrow) in the foetal skin HFs ((a) from Google images) but not morphologically distinguishable (arrow) in the adult skin HFs (b). (c) and (d) AU pedicles. Note that pedicles are the longest (arrow; hence, the PP is the largest in area) in the first year of deer's life (c) but totally disappear (arrow) in the mature stags (d).

Likewise, when a small population of AP cells was labelled with LacZ gene prior to antler generation, *β*-galactosidase-positive cells could be detected in every mesenchymal tissue component (except for skin dermis) in the subsequent developed AU including reserve mesenchyme (Figure 4(d)), precartilage (Figure 4(e)), cartilage (Figure 4(f)), and lamellabone (Figure 4(g)). Interestingly, the bulge is a very prominent structure of HFs in foetal skin (Figure 5(a)), but it becomes smaller with age and is not morphologically distinguishable (Figure 5(b)) in the HFs of adult skin [4]. Likewise, the pedicles are the longest in the first year of a deer's life and they contain the greatest number of periosteal cells in the PP (Figure 5(c)). The length of the pedicle is progressively shortened each year with each cycle of regeneration and, in older stags, the pedicle structure is absent (Figure 5(d)) [45]. Surprisingly, the disappearance of the tissue that contains HF/AU stem cells in adult animals does not abrogate or influence subsequent regeneration of the cyclic part of each respective organ. This implies that the stem cells in the HF “invisible bulge” or the cells residing in the marginal periosteum surrounding a pedicle have the ability to self-renew and replenish the progenitor pool and give rise to transient amplifying cells for the cyclic regeneration of each organ.

Overall, organogenesis and cyclic regeneration of HF and AU are both stem-cell-based processes. HF stem-cells are located in the bulge and AU stem cells in the AP/PP, respectively. Notably, both HF and AU stem cells express key embryonic stem-cell markers in addition to their respective tissue-specific stem-cell markers and can be induced to differentiate into multiple cell lineages in vitro [37, 45].

## 4. Dependency on Epithelial-Mesenchymal Interactions

In order for stem cells to self-renew and replenish the pool of stem cells for subsequent rounds of regeneration, they must be located in their niche and interact with the other cell types [49]. Amongst these cell types, the most important are for the HF are the DP cells and for the AU the epidermal cells. Each represents a type of tightly coordinated interaction between the epithelium and mesenchyme (E-M interaction) that is responsible not only for organogenesis, but also for subsequent cyclic regeneration.

**Figure 6:**
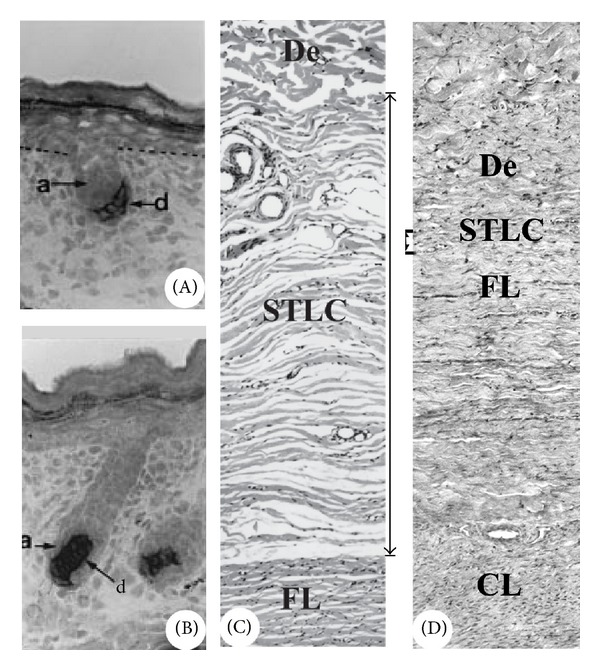
Tissue close association in organogenesis. (A) and (B) Reproduced with permission from [5, Figures 2G and 2M, resp.]. HFs in different developmental stages to show the close association between the mesenchyme-derived DP (d) and the epithelium-derived germ/matrix cells (A). Note that at stage 2 of HF formation, the hair peg is capped by DP cells (A), and at the bulbous peg stage, the DP cells are wrapped by HF matrix cells (B). (C) and (D) AUs in different developmental stages to show the close association between the AP-derived tissue (FL, fibrous layer of AP) and the overlying skin (De, skin dermis) prior to first antler initiation. Note that at the early pedicle stage, the two tissue types are separated by a very wide and loose layer of connective tissue (SLCT, (C)), but at the late pedicle stage, the two tissue types become closely associated (D). CL; cellular layer of AP.

During early organogenesis of the HF, the hair germ layer becomes visible as an epidermal thickening and the dermal fibroblasts immediately below the thickened germ layer start to change their orientation. As an HF elongates, the underlying dermal fibroblasts gradually aggregate to form a cap-like structure that abuts closely to the distal end of the hair peg (Figure 6(A)). At the bulbous peg stage, the condensed dermal fibroblasts (now called DP) are completely enclosed by the epithelium-derived hair matrix cells (Figure 6(B)). During the entire course of mouse HF organogenesis, the mesenchyme-derived DP and the epithelium-derived germ/matrix cells remain closely associated with each other [5]. This phenomenon strongly suggests that the DP is involved in HF organogenesis through interacting with HF germ cells.

Prior to the development of an AU, the AP (mesenchyme-derivative) and the overlying skin (particularly the epithelium-derived epidermis) are separated by a wide and loose layer of subcutaneous connective tissue (Figure 6(C)). A pedicle forms when AP cells are triggered by endocrine factors (such as androgens) to proliferate and differentiate [15, 50]. The expansion of antlerogenic tissue progressively creates a mechanical tension to the overlying skin, which causes compression of the interposing subcutaneous connective tissue between them. The initiation of antler growth from a developing pedicle does not start until the interposing layer has been substantially compressed and stretched to become essentially a thin strip (reduced to approximately a 20th of the original thickness), which brings the antlerogenic tissue and the overlying skin in close apposition (Figure 6(D)). This intimate association has been suggested to be the prerequisite for the establishment of the E-M interactions, which is required for antler organogenesis [17, 51, 52].

**Figure 7:**
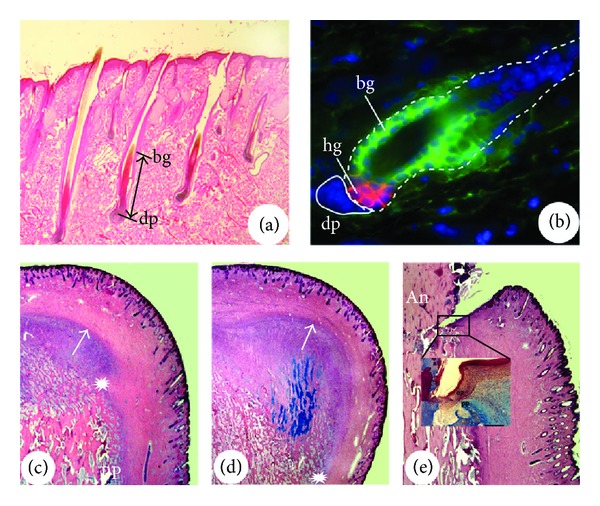
Tissue close association in cyclic regeneration. (a) and (b) Reproduced with permission from [20, Figure 1B]. HFs in different developmental stages. Note that at the late anagen (a), the DP (dp) of the bulb has the longest distance from the bulge (bg), but at the telogen (b), the DP (dp) is closely attached to the bulge- (bg-) derived hair germ (hg). (c)–(e) AUs in different developmental stages. Note that at the mid wound healing stage (c), a growth centre is formed by proliferating and differentiating distal PP cells and expansion of the centre starts to push the overlying skin away (arrow points the growth direction) from the distal PP region (asterisk); at the late wound healing stage (d), the centre pushes the overlying skin (now is velvet in nature) even further away (arrow points the growth direction) from the distal PP region (asterisk); and at the hard antler (An) phase (e), pedicle skin epidermis (ep) seals the broken end of the dermis and rests on the distal end of PP ((e), Inset).

E-M interactions are also periodically reactivated throughout adult life as components of the developmental program reoccur during the onset of each cyclic regeneration of HF or antler. In the early phase of anagen in HF, the DP is progressively separated from the bulge due to rapid expansion of the hair germ-derived cell mass, until the establishment of the mature anagen follicle (Figure 7(a)). At the anagen/catagen transition, HF matrix cells are subjected to apoptosis and the DP retracts upward towards the bulge along with the dying epithelial strand. Throughout the entire telogen phase, the DP directly abuts with the base of a bulge (Figure 7(b)), such that interactions between these two components would be facilitated in preparation for the next round of HF regeneration.

During early antler regeneration, the skin (particularly epidermis) that covers the posterior (the site where the main beam will form) and anterior (brow tine) edges of a pedicle stump is rapidly displaced from the stem-cell niche, the distal region of PP (Figures 7(c) and 7(d)), due to the rapid expansion of the PP-derived cell mass. Subsequently, the growth centres of the main beam and brow tine are established by the transient amplifying cells of PP origin, and growth of each centre pushes the skin farther away through neogenesis of velvet skin to accommodate the expanding tissue mass. After full antler regeneration, the process of velvet shedding interrupts the integrity of the skin at the site between the pedicle and antler and exposes the distal end of pedicle skin and PP. The epidermis of the pedicle skin rapidly expands to seal the wound. During the entire hard antler (resting) phase, the distal end of the pedicle skin epidermis firmly abuts its dermis and the PP and acquires some velvet skin features (Figure 7(e)) prior to antler regeneration [17, 32].

To experimentally confirm that the DP in HF or the epidermis in AU is indispensible to the organogenesis and regeneration of each respective organ, both tissue deletion and transplantation methods have been employed. Unexpectedly, the tissue deletion approach was ineffective in preventing regeneration of both HF (rat whiskers) and AU. This is because the removal of the DP fails to stop HF organogenesis or regeneration, as the cells from the remnant outer root sheath and its adherent mesenchymal layer can compensate for this loss [53]. Likewise, by the removal of the skin overlying the AP [2, 54] or enveloping the PP [2], an antler would still generate/regenerate as cells from the skin wound margin eventually heal the wound and reestablish interactions with the closely associated antlerogenic tissue.

**Figure 8:**
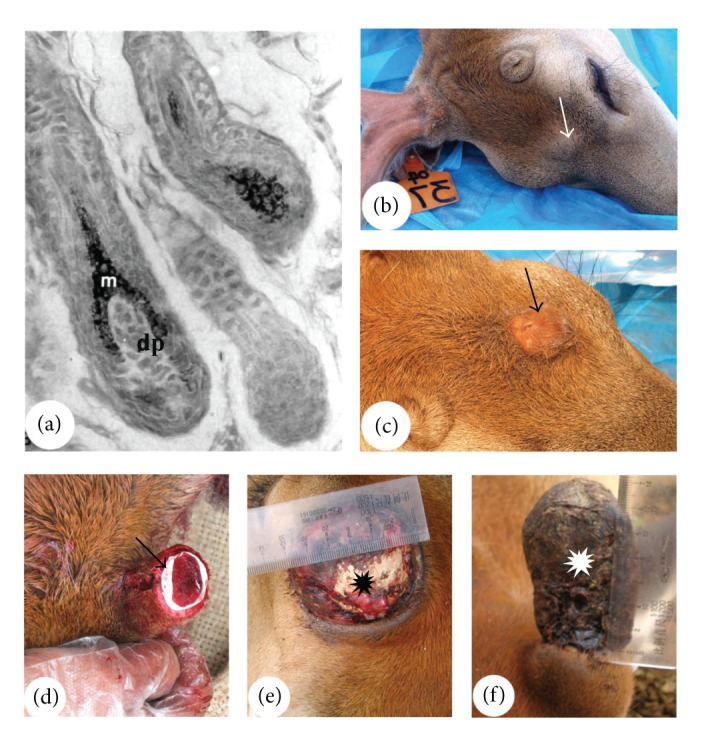
Confirmation of specificity of the interactive tissue types in the organogenesis and regeneration through transplantation. (a) Reproduced with permission from [21, Figure 3B]. HFs that were generated by introduction of DP cells subepidermis of the footpad skin (m; matrix cells; dp; dermal papilla). (b) and (c) Reproduced with permission from [22, Figures 4D and 4F, resp.]. Bumps that were formed from the subcutaneously grafted AP. Note that when an impermeable membrane was inserted between the grafted AP and the overlying skin, no skin transformation nor antler growth occurred (arrow, (b)), but when the impermeable one was replaced with a semipermeable one, skin transformation and antler formation took place (arrow, (c)). (d)–(f) Reproduced with permission from [10, Figures 1E, 3F, and 2B, resp.]. Membrane insertion (arrow) between the PP and the enveloping skin (d). Note that when the membrane was inserted at the loosely attached region (proximal side of a pedicle), no antler regeneration (asterisk) occurred (e); but at the closely associated region (distal side of a pedicle), a skinless antler (asterisk) regenerated (f).

In contrast to the approach of tissue deletion, experiments involving the transplantation of cells or tissue have convincingly demonstrated that the DP is the key tissue component for the initiation of HF. Reynolds and Jahoda [21] reported that DP cells from the rat pelage follicle can successfully interact with epidermis of the footpad skin to initiate HF organogenesis and external hair growth (Figure 8(a)). Furthermore, the grafted human DP can induce the skin of nude mice to form new fibre-producing follicles [55]. Therefore, the skin from the rat footpad or nude mice cannot grow hair because it does not contain competent DP cells, which are necessary for HF stem-cell induction of organogenesis and regeneration.

In the case of AU, the importance of communication between the two tissues was demonstrated by inserting a thin membrane between the skin and periosteum to show the dependency on skin for antler organogenesis [22] and regeneration [56]. When a piece of impermeable membrane was inserted between the grafted AP and the overlying skin prior to AU formation, antlers did not develop (Figure 8(b)), whereas, when a semipermeable membrane (with 0.45 um pore size) was substituted, antlerogenesis eventually occurred (Figure 8(c)) although the onset was delayed for about a year. When an impermeable membrane was inserted between the PP and pedicle skin (Figure 8(d)) in the proximal region (the two interactive tissues are loosely associated in this region) of a pedicle stump, antler regeneration failed to occur because of the absence of an E-M interaction (Figure 8(e)). However, if the impermeable membrane is inserted in the distal region (the two interactive tissues are tightly associated in this region), antler regeneration occurred (though without skin, Figure 8(f)) because the E-M interactions were already established. These results suggest that stem-cell-mediated antler regeneration requires an interaction with skin [56]. Therefore, the membrane insertion experiments have not only confirmed that both antler generation and regeneration depend on E-M interactions, but also demonstrated that these interactions are essentially realised through the exchange of small diffusible molecules.

**Figure 9:**
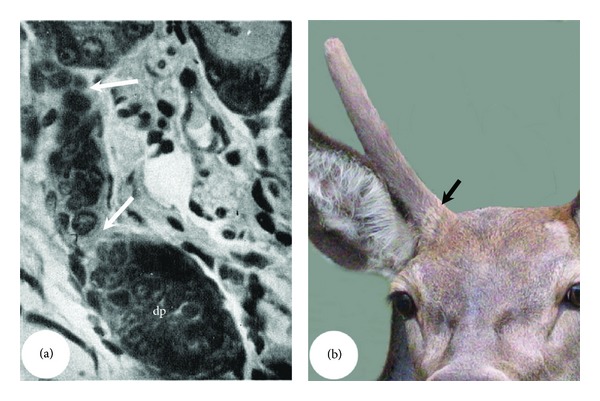
Examples showing the importance of the interactive tissue types coming together for organogenesis. (a) Reproduced with permission from [23, Plate 1 Figure 2]. Broken HF epithelial strand (arrows) in a hairless mouse skin that failed to bring the DP (dp) upward towards the bulge. (b) Perennial antler grown by a female deer. This type of antlers is not subject to annual regeneration cycles as they do not shed their velvet; hence, the epidermis of a pedicle skin cannot come to abut directly on the PP.

Further examples illustrating the importance of this E-M interaction are tissue transplants into hairless mice, and the antlers grown by castrated male and female deer. In mutant hairless mice [57, 58], the development of the first hair is normal up to the formation of an epithelial strand connecting the bulge of the permanent component and the DP of the cyclic component in catagen. However, the strand then fails to shorten and becomes constricted and interrupted in places (Figure 9(a)). Consequently, the DP remains separated from the bulge and no subsequent regeneration occurs, resulting in the development of a “hairless” phenotype. Antlers grown by female deer (Figure 9(b)), either naturally [59] or artificially induced [60], and by male deer castrated in the antler growing phase [61] remain permanently viable and do not undergo cyclic regeneration. This may be due to a failure to establish the interactions between the pedicle skin epidermis and the PP, as these two components are physically separated in these permanently viable antlers. This hypothesis is supported by the observation that mechanically breaking the integrity of the skin (by cutting off a viable velvet antler at the junction of an antler and a pedicle) triggers a new cycle of antler regeneration [25]. This observation reinforces the importance of the close association and interaction between antler stem cells (PP cells) and the other cell types (pedicle skin cells) for antler regeneration.

In summary, generation and regeneration of both HF and AU rely on interactions between the stem cells of each organ (bulge cells in HF and periosteal cells in AU) and the other key cell types (DP cells in HF and skin cells in AU). To enable each process to occur, the two interactive tissue types must be intimately juxtaposed although the means through which this close association is achieved is different for each tissue: for HF by emerging together in organogenesis and by the destruction (via apoptosis) of the intervening suprabulbar strand during regeneration; and for AU by compression of the loose connective tissue layer during AU generation and by breaking the integrity of skin and PP through velvet shedding in antler regeneration. Induction of the DP in early anagen activates some of bulge stem cells, leading to the proliferation of these cells to form the epithelial-derived cyclic component of the HF. Feedback from the activated and rapidly proliferating stem cells drives the DP to undergo characteristic changes in its volume, histological appearance, and composition of the basement membrane [62, 63]. Likewise, induction from the AP turns the typical deer scalp skin epidermis into antler velvet and feedback from the transformed velvet epidermis (possibly through dermis) drives the cells of AP derivative to rapidly proliferate to form an antler [45]. In each case, the mesenchymal cells are the inducer and the epithelial cells are the responder, irrespective of whether epithelial or mesenchymal cells are the stem cells initiating the process although, in HF, the inducer (DP) does not physically participate in the organ (hair) formation, while, in AU, the inducer is also the cell type that gives rise to the organ (antler).

## 5. Dying for Stem-Cell Recruitment

Cyclic regeneration of HF has evolved in mammals as a means for replacement (moulting), camouflage, temperature regulation, or social and sexual signalling [64]. Likewise, deer have adopted similar mechanisms for antler regeneration to prevent growing antlers from freezing if deer happen to inhabit temperate zones to repair broken tines and to maintain in proportion to body size [65]. To enable these organs to regenerate, they cease growth after reaching maximal size and eventually enter a regressive phase ultimately leading to the reactivation of dormant stem cells in the niche to initiate a new cycle. Currently, there are two hypotheses to explain the phenomenon of growth cessation in HF. The first is that the production of hair fibres ceases because the matrix cells have exhausted their proliferative capacities [66]. The second is that the HF stem cells may continuously generate new matrix cells, with the production of hair fibres ceasing at a preprogrammed point that depends on many factors including the environment, follicle type, age, sex, and species [18].

The rationale for the first hypothesis is that the proliferative capacity of matrix cells is determined at the initiation of a new hair cycle and that new matrix cells are not generated throughout the entire growth phase. Because transient amplifying cells have a limited potential to proliferate and because the majority of matrix cells are actively involved in continuous replication [4], they eventually exhaust their proliferative capacity and undergo terminal differentiation. The second hypothesis is based on the results from clonal studies and from studies in which the cyclic component of the mouse HF was transplanted [18]. In those studies, the matrix keratinocytes were demonstrated to have the potential to replicate beyond that normally achieved. Therefore, the final regeneration length of an HF must be controlled by extrinsic factors, rather than the limited potential of matrix cells to proliferate. The finding that an epidermally derived, telogen-specific molecule can inhibit HF growth [67] lends support to this view. Such a factor could be considered to be an epidermal chalone. A number of studies also show that prolactin, an endocrine factor, is implicated in controlling seasonal HF cycles [68].

It is well established that regenerating antlers cease growing due to extensive calcification caused by the sharp increase in concentrations of circulating androgen hormones [69]. Therefore, cessation of antler growth can be better explained by the second hypothesis for HF, that is, extrinsic factors controlling the process. Interestingly, a regenerating antler does not have the potential to grow much further when the source of androgens is removed by castration at the late antler growth phase although the antler remains permanently viable during the life of a deer [61]. In view of this finding, Li et al. [45] suggested that when growth of a velvet antler is terminated by extensive calcification, the mesenchymal cells in the antler growth centre have almost exhausted their ability to proliferate. Because antler mesenchymal cells have a limited potential to proliferate [12], the replicative potential of these cells, while terminated by calcification, is almost exhausted when the growth of antlers nears completion. This hypothesis is the combination of the first and the second hypotheses for HF.

**Figure 10:**
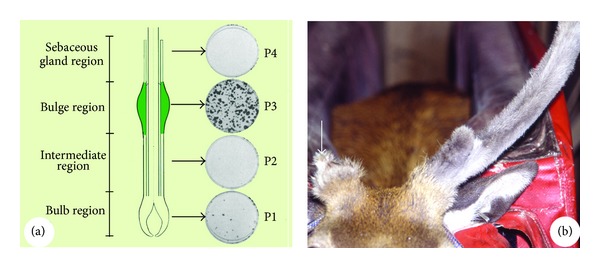
Demonstration of the difference in proliferation potential between the stem cells and the transient amplifying cells. (a) Reproduced with permission from [24, Figure 2]. Difference in clonogenicity between the bulge cells and bulb cells of HFs. Note that the bulge (P3) contains 95% of the clonogenic keratinocytes, whereas the bulb (P1) contains 5%, and no clonogenic keratinocytes were detected in the rest of the portions (P2 and P4). (b) Reproduced with permission from [25, Figure 3C]. Difference in regeneration potential between the pedicle stump (stem cells) and the antler remnant (transient amplifying cells). Note that there was no difference in size between the 1st and the 4th set antlers regenerated from the pedicle stump; in contrast, the 4th set antler regenerated from the antler remnant was a small aborted one (arrow).

The following experiments provide evidence that there is a marked difference between stem cells and the transient amplifying cells of HF or AU in their ability to proliferate. Cotsarelis et al. [4] found that stem cells are enriched in the mouse HF bulge but not elsewhere in the follicle including the bulb. Kobayashi et al. [24] reported that the bulge region of rat vibrissa contains 95% of the clonogenic keratinocytes present in an anagen rat follicle, whereas the hair bulb contained the remaining 5% (Figure 10(a)). These results demonstrate that HF stem cells located in the bulge have the potential to proliferate more extensively than those found in the cyclic regeneration component including the bulb.

The claim that transient amplifying cells in the AU have a limited potential to proliferate is supported by our previous experiment where perennial living antlers were created by castration [25]. In that experiment, two types of stumps were generated by removing the perennial antlers at either the junction (to expose PP cells to the epidermal cells) with the pedicle (pedicle stumps) or 2 cm above the junction (antler remnants) to expose the transient amplifying antler cells to the epidermal cells. After removal of antlers at the level of the pedicle in five consecutive cycles, no significant difference (*P* > 0.05) in antler length was detected between the first and the fifth sets. In contrast, the regenerative potential of the antler remnants was significantly decreased with successive cycles of removal and regeneration, and regrowth was almost totally exhausted after the third cycle when only small antlers were formed (Figure 10(b)).

In summary, the transient amplifying cells in both the HF and the AU have a limited potential to proliferate. To enable a larger or differently shaped appendage to form/regenerate, stem cells must be recruited, which is achieved through destruction of the cyclically regenerated component in order to bring the reactive cell types into the proximity with the stem-cell niche.

## 6. Systemic and Local Controls

HFs and AUs have evolved to protect their hosts, as insulation and camouflage for hair and as weapons and visual display for antlers. Hence, each phase of their regeneration cycle must be synchronised with season. Thus, thick fur must be grown for winter and hard antlers for autumn rutting (a period of heightened sexual activity). Synchronisation is largely entrained by photoperiod and temperature. By artificially manipulating photoperiod, the frequency and amplitude of the growth cycles of these appendages can be profoundly affected. For example, thick winter or thin summer coats of mink can be readily achieved by artificially altering day length [70]. Up to four antler growth cycles can be produced in one calendar year if deer are exposed to four rounds of increasing and decreasing photoperiods in the same 12-month interval [71]. It is now well established that these environmental cues are transduced to HFs [72] or to antlers [73, 74] via the pineal and the hypothalamus-pituitary route involving gonadal, thyroid, and other endocrine hormones especially melatonin, testosterone, and prolactin.

### 6.1. Endocrine Factors

Of the endocrine factors, androgen hormones are reported to be the most important for regulating the types of fibre produced by HFs in some species, including humans [64, 75]. Changes in hair type from fine unpigmented vellus follicles to thick pigmented terminal hair on the face, chest, and upper pubic triangle of adult males occur during periods of increasing concentrations of androgens in blood [64]. Similarly, AUs are secondary sexual characteristics of male deer whose organogenesis is triggered by the elevated concentrations of circulating androgens when deer approach puberty [15, 76].

The connection between human hair or deer antlers and the testis was first noticed by Aristotle over 2000 years ago: boys castrated before puberty do not grow sexual hair (cited by [75]) and prepubertally castrated deer do not grow antlers (cited by [77]). Studies in recent history show that exogenous testosterone can stimulate the growth of beards in eunuchs [78], while, conversely, patients with complete androgen insensitivity syndrome (testicular feminization), that is, lacking functional androgen receptors, do not develop a beard, maxillary, or pubic hair [79]. Similarly, administration of exogenous testosterone can successfully stimulate the prepubertally castrated deer to grow AUs [15].

Clearly circannual variation in the growth of hair on the scalp, face, and thigh in human has been linked to seasonal changes in concentrations of androgens in blood [80]. For example, growth of beards was slower in January/February and the rate increased steadily to reach a peak that was about 60% higher in July, and a similar pattern was observed for growth of hair on the thigh [81, 82]. The most convincing example demonstrating the influence of androgen hormones on circannual variation in HFs is the seasonal change of growth of the mane in adult male red deer [83]. The long hair on the deer mane is at least twice the length of winter coat hair and develops from August until December [84], coinciding with the increase in concentrations of plasma testosterone, whereas the growth of short hair on the neck occurs in spring and summer, a period coinciding with low concentrations of circulating testosterone [33].

The annual growth of antlers (see the Ontogeny Section in this review) is strictly under the control of circulating androgen hormones, particularly testosterone. Each year, hard antlers are cast from their pedicles when concentrations of testosterone decrease to a certain threshold. Wound healing over the pedicle stump and antler regeneration take place while concentrations of testosterone remain low. Antler growth gradually ceases due to an increase in calcification caused by the rapid increase in concentrations of circulating testosterone and this is accompanied by shedding of the velvet skin [50, 85].

Because androgen receptors are only found in the DP cells in HFs [86, 87], it has been claimed that androgens regulate growth and development of HFs through directly acting on the DP cells and then indirectly on the other HF cell types [64]. Likewise, androgen receptors are only detected in the AP cells in AUs [88, 89]. Li et al. [45] proposed that androgens control AU development through directly acting on the antler stem cells. Surprisingly, neither DP cells [90] nor AP cells [91] are stimulated to proliferate in vitro in response to androgen hormones.

### 6.2. Paracrine Factors

The growth of the HF and AU is also regulated by a number of potent growth factors. Among these factors, insulin-like growth factor 1 (IGF-1) has been particularly important for the growth of these organs with dose-dependent mitogenic effects on both HF [92] and AU stem cells [91] in vitro.

It is currently understood that androgens act on HFs via androgen receptors within the DP cells and trigger the expression of hormone responsive genes. This then alters the paracrine factors produced by the DP cells which regulate the growth and activity of other cell types in the human HF. These paracrine factors could be soluble mitogenic factors or extracellular matrix components [93]. We postulate that interactions between antler stem cells and the associated skin cells for initiating antler generation and regeneration are realised through exchange of diffusible molecules. In support, the interposition of an impermeable membrane between these two cell types prevents the initial growth and regeneration of antlers, whereas a semipermeable membrane does not inhibit but, rather, delays the process [22]. The identity of these paracrine molecules remains unknown at present.

The paracrine factors responsible for mediating the communication between the epithelium and mesenchyme tissues during organogenesis of HF and regeneration include members of the Wnt/wingless family and the hedgehog family and of the TGF-*β*/BMP, FGF, and TNF families [94]. Canonical Wnt/*β*-catenin signalling provides the master switch for determining the fate of HFs because expression of the Wnt inhibitor Dkk1 or lack of epidermal *β*-catenin resulted in the lack of induction of hair follicle growth [95, 96]. Conversely, forced expression of a stabilized form of *β*-catenin causes an enhanced formation of placodes, and epidermal keratinocytes globally adopt an HF fate [97, 98]. Likewise, it has been reported [99] that the most intense *β*-catenin staining was detected in dividing, undifferentiated mesenchymal cells in the growth centre of early regenerating antler bud. When the canonical Wnt pathway was inhibited at the level of Lef/TCF, the number of antler cells decreased as a result of increased apoptosis. Activation of the Wnt pathway inhibited alkaline phosphate activity (a marker of antler progenitor cell differentiation) of these antler cells. Therefore, *β*-catenin plays an important role in the regulation of antler progenitor cell survival and cell fate; and hence, Wnt signalling is important for antlerogenesis.

Sonic hedgehog, an epithelial placode cell factor, signals to the underlying mesenchyme during HF organogenesis to form the dermal condensate which subsequently gives rise to the DP [100]. This factor is also expressed in antler tissue [101, 102]. Vascular endothelial growth factor, VEGF, is a major regulator of angiogenesis [103] and is expressed in both human DP cells [104] and antler cells [105], which is not surprising given that both tissues are highly metabolic and require a good blood supply.

Maintenance of stem cells is ensured by slow cycling, which is controlled by low levels of c-myc in mammalian HFs [106]. Antler stem cells (AP and PP cells) also express c-myc [48], so this gene may also be required for the maintenance of antler stem cells. Both the AP and mouse HF bulge stem cells express S100A4, a calcium-binding protein [48, 107]. Interestingly, only the hair germ cells, but not the bulge cells, are involved in plucking-induced onset of a new hair cycle, and the hair germ cells do not express S100A4. Likewise, only the PP cells are responsible for antler regeneration, but the PP cells do not express S100A4. In this regard, the PP cells in antler regeneration act as the hair germ cells for regeneration of HFs.

## 7. Connections during Antlerogenesis

**Figure 11:**
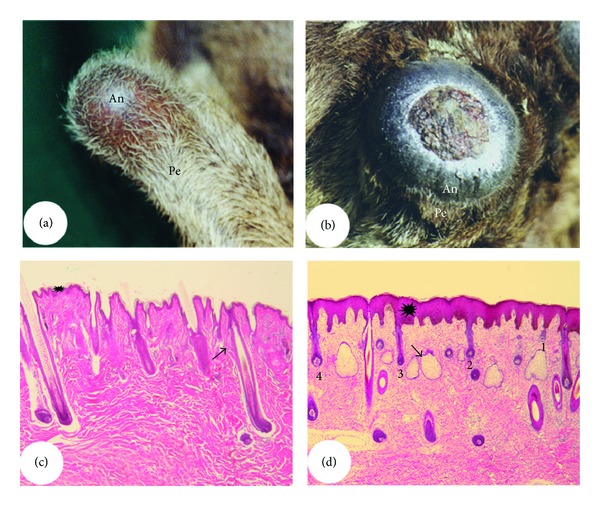
Differences between pedicle skin and antler velvet. (a) and (b) Pedicles and incipient generating (a) or regenerating (b) antler from a red deer. Note that comparing to pedicle skin (typical scalp skin), hairs of antler velvet are shorter, thinner, more sparsely populated, and growing out nearly at right angles to the skin surface. (c) and (d) Histological sections of pedicle skin (c) and antler velvet (d). Note that comparing to the pedicle skin, antler velvet has thickened epidermis (asterisk), large multilobed sebaceous glands (arrow), and neogenesis of HFs at different developmental stages (1, 2, 3, and 4) but has lost the arrector pili muscle and sweat glands.

Both generation and regeneration of the AU have been considered to be unique zoological phenomena [2, 45, 108, 109], partially because these processes occur in postnatal life and involve the transformation of the mature scalp skin into velvet skin. The hairs on velvet are shorter, thinner, more sparsely populated, and growing at right angles to the skin surface (Figures 11(a) and 11(b)). Histological examination [17] shows that this change in skin type includes an almost 10-fold thickening of the epidermis, a loss of arrector pili muscles and sweat glands, a gain of large bi- or multilobed sebaceous glands, and neogenesis of HFs (Figures 11(c) and 11(d)). It is not clear whether HFs in velvet skin possess a bulge, as this structure is not discernable at a histological level. Arguing against the existence of the bulge is the observation that HFs in velvet skin are not subjected to cyclic regeneration (hence, the presence of stem cells may not be required) as velvet skin is short-lived (<100 days). In addition, HFs in velvet skin do not possess arrector pili muscles while the bulge is normally located at the site where arrector pili muscle attaches to the outer root sheath. A counter argument that favours the existence of the bulge in HFs of velvet skin comes from the observation that transplanted velvet elsewhere on the deer body must have undergone cycles of regeneration to have survived for years without shedding [2]. A simple immunostain using a bulge stem cell marker, such as SOX9 [41], should clarify whether or not the bulge is present in HFs of velvet skin.

**Figure 12:**
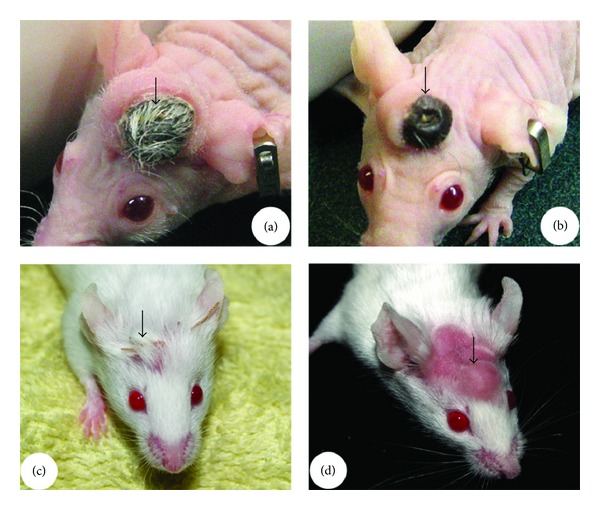
Xenotransplantation of AP. (a) and (b) Reproduced with permission from [26, Figures 2B and 2F, resp.]. AP was transplanted with a piece of deer scalp skin (sutured together) onto the head of a nude mouse (arrow, (a)), and, subsequently, the scalp skin was transformed into antler velvet (arrow, (b)). (c) and (d) AP was subcutaneously transplanted onto the head of a normal laboratory mouse (arrow, (c)), and, subsequently, the developing AP tissue turned the overlying mouse skin into an essentially bold one (arrow, (d)).

A histological examination indicates that one of the most obvious effects of AP/PP induction to the overlying skin during AU generation or antler regeneration is the formation of miniaturised HFs (velvet skin HFs produce the thinnest hairs on deer [110]). This histological finding can be confirmed by subcutaneously transplanting AP tissue to induce ectopic antler formation [19, 42]. During the initiation of ectopic antler formation, rapid growing of the grafted AP tissue creates mechanical tension to the overlying somatic skin, which drives neogenesis of skin with a fine sparsely populated hair characteristic of velvet skin (Figure 3(b)). To determine whether systemic factors are involved in the transformation of skin (as HF miniaturisation can also be induced by circulating androgen hormones, such as that occurs in alopecia [80]), we carried out a xenotransplantation experiment [26] to subcutaneously graft the tightly bound AP and deer scalp skin (sutured together) onto the head of a nude mouse (Figure 12(a)). The loose connective tissue and the associated partial dermal layer were removed from the transplanted deer skin to just below the HFs. Transformation of skin to antler velvet occurred on the head of a mouse around one and half months after the transplantation (Figure 12(b)). The results of this experiment not only demonstrate that factors solely derived from AP are sufficient to induce transformation of the skin, but also show that the removed partial dermal tissue is not required for the induction. To test whether induction is species-specific, we subcutaneously transplanted a small piece of AP onto the head of a conventional laboratory mouse (Figure 12(c)). The unpublished results were surprising, not because the grafted AP developed into a nodule with an appreciable size in a normal mouse (possibly AP tissue is immune-privileged as there was no obvious immune-rejection), but because the developed AP tissue converted the overlying mouse skin into a hairless phenotype (Figure 12(d)). Therefore, AP-derived factors may have the ability to influence HFs from a wide range of hosts. Overall, skin neogenesis that is driven by rapid forming AP tissue accompanies with miniaturised HFs: reduced HF density and size, lacks of arrector pili muscle and sweat glands, but enlarged development of sebaceous glands.

**Figure 13:**
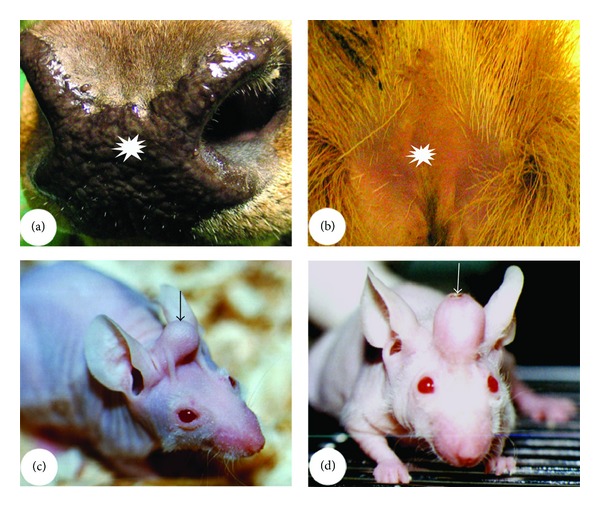
Skin type and antlerogenesis. (a) Deer nose snout (asterisk). (b) The ventral surface of a deer tail (asterisk). (c) and (d) Nude mouse skin (arrow). Note that all these three hairless skin types are incompetent to interact with the grafted AP to initiate antler formation; even if wounding (arrow) was carried out, no antler growth occurred (d).

If the AP restricts the development of HFs during antlerogenesis, what would happen if the skin associated with the AP is hairless? When transplanted underneath the hairless skin, such as the snout of a deer's nose (Figure 13(a)) or the ventral surface of a deer tail (Figure 13(b)), the AP failed to initiate ectopic antler formation from these grafted sites [46]. Interestingly, when the AP is transplanted under the skin of nude mice [111], a common animal model for hairless skin, formation of ectopic antlers does not occur although sizable pedicle-like nodules were formed (Figure 13(c)). Even if those ectopically developed nodules in nude mice are apically wounded in a manner mimicking the casting of antlers [60], no initiation of antler growth was observed (Figure 13(d)). The skin overlying those nodules remained loose even when enlargement of the nodule caused the skin to be significantly elevated, indicating that hairless skin is incompetent to interact with the underlying grafted AP. Alternatively, nude mouse skin does contain hair follicles, but only species-specific hair follicles can serve a role in antler formation. Therefore, HFs may supply the key skin component mediating interactions between the AP-derived tissue and the skin, and the specific feedback from the HFs to AP is essential for antlerogenesis to take place.

**Figure 14:**
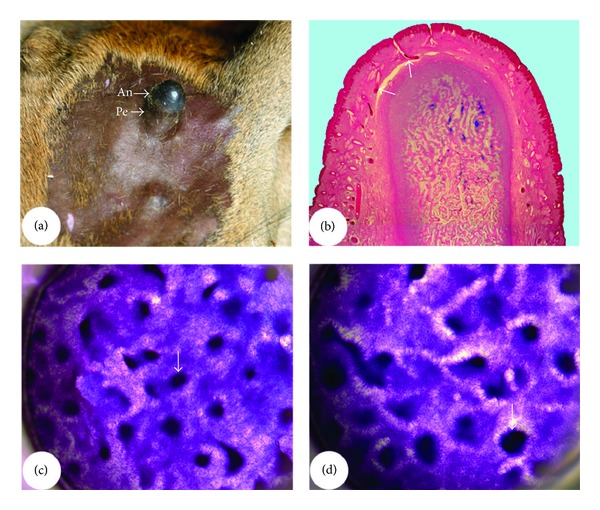
HF involvement in antlerogenesis. (a) and (b) Intradermal transplantation of the minced AP (delivering AP tissue directly under the HFs). (a) The 1/8 AP successfully induced ectopic pedicle (Pe) antler (An) formation (subcutaneous transplantation requires at least half of the AP tissue). (b) Histological section shows that the lower parts of HFs in the apical velvet skin were bent away (arrows) from the direction of AP tissue growth. (c) and (d) AP cells were cocultured with the DP cells of pedicle skin HFs using a cell culture insert. Note that the size of DP cell aggregates significantly reduced in the coculture (arrow, (c)) comparing with the singular DP cell culture (arrow, (d)).

Direct confirmation as to whether HFs truly mediate the interactions between the AP and skin during antlerogenesis, such as ablation of the HF to see if the skin still can participate in antlerogenesis, is not always practical. An alternative approach to test this hypothesis was to deliver minced AP tissue directly under the bulbs of HFs [47] to determine if physically placing antler stem cells and the putative reactive tissue together would facilitate initiation of antler formation. To achieve this, an intradermal pocket was firstly made through a horizontal incision in the skin directly under the HFs. The results strongly support the view that HFs are required to mediate antlerogenesis because only an eighth of an AP tissue implant (a whole piece of AP is about 25 mm in diameter and 2-3 mm in thickness in red or sika deer) was needed when delivered in this manner (Figure 14(a)), whereas at least half of an AP tissue implant is required to induce growth of ectopic antlers when grafted under the skin. Interestingly, the lower parts of some HFs in the apical skin of the antlers formed from the intradermal pocket approach did not grow into the AP-derived tissue or were pushed upward, instead were bent away from it, possibly caused by the mechanical force which is created by the underneath AP tissue expansion (Figure 14(b)). Furthermore, when cocultured in vitro using a tissue culture insert, AP cells on one side of an inserted membrane significantly reduce the size of DP cell aggregates on the other side (Figure 14(c)) compared to control cells (facial periosteal cells) (Figure 14(d); unpublished). Because it has been well established that the thickness of an HF/hair corresponds to the size of a DP, the effects of AP on miniaturisation of HF/hair may be mediated through the DP [112–115].

In summary, antlerogenesis depends on interactions between AP/PP-derived tissue and the overlying skin. The available evidence indicates that these interactions are mediated by the HFs residing in the AP/PP associated skin. On the one hand, antlerogenesis requires the presence of HFs, but on the other hand, antlerogenesis produces skin that adorns with miniaturised HFs.

## 8. Concluding Remarks

In this review, we have made comparisons between HFs and AUs—two seemingly unrelated mammalian organs. HFs are tiny and are concealed within skin, whereas AUs are gigantic and are grown externally for visual display. However, these two organs share some striking similarities (Table 1). Both organs consist of permanent and cyclic/temporary components and undergo organogenesis and stem-cell-based cyclic regeneration. Stem cells of both organs reside in the permanent part and the growth centres are located in the temporary part of each respective organ. Organogenesis and regeneration of both organs depend on E-M interactions. Establishment of these interactions requires stem cells and reactive cells (DP cells for HFs and epidermal cells for AUs) to be juxtaposed, which occurs through destruction of the temporal part to bring the respective reactive cells into close proximity to the stem-cell niche. Therefore, these two organs share a similar ontogenetic developmental process.

Since HFs adorn the integument of almost every mammalian species including humans, their organogenesis and cyclic regeneration have been intensively investigated and some of the molecular mechanisms underlying these developmental processes have been elucidated [116]. In contrast, AUs are solely grown by male deer (except in reindeer), and research into their molecular mechanisms is still at the preliminary stage. However, the structure and development of AUs and the HFs in velvet skin have unique attributes that could offer a fascinating new model system for further deciphering the underlying mechanism for the formation of an HF. Therefore, we believe that investigators from both fields could greatly benefit from a comprehensive comparison between these two organs.

### 8.1. For the Benefit of Antler Biologists

Resistance of stem cells in the mouse HF bulge to DNA-damage-induced cell death is a consequence of higher expression of the antiapoptotic protein Bcl-2, enhanced DNA repair activity, and the rapidly attenuated activity of p53 [117]. Expression of Bcl-2 is also observed in the mesenchymal tissue of antler (transient amplifying cells [118]); is this gene also expressed in the AP and/or PP tissue? If it is, this gene may also be important for the maintenance of antler stem cells.

In HFs, telogen can be divided into a phase that is refractory to HF growth stimuli and that is characterized by upregulation and activation of BMP2/4 and a competent phase in which bulge stem cells become highly sensitive to anagen-inducing factors [119]. In the competent phase of regenerating HFs, BMP signalling is turned off while Wnt/b-catenin signalling is turned on to reach its optimal activity in early anagen. How about AUs? The transition from velvet to hard antler can also be divided into refractory and competent phases to mitogenic factors. Do the factors that operate in the HFs also function in AUs?

### 8.2. For the Benefit of HF Biologists

Formation of the pedicle is independent of the E-M interactions and is solely triggered by the increase in concentrations of circulating androgen hormones [15]. When the pedicle reaches the species-specific height, AP-derived mesenchymal tissue becomes closely associated with the overlying skin and the two tissue components are then able to interact and initiate growth of the antler [17]; that is, anything formed through the E-M interactions during the initial AU generation will be destroyed and rebuilt in subsequent cycles of antler growth. How does this compare with HFs? Morphologically, at the early stage (before development of the HF peg), no obvious aggregation of dermal cells can be detected under the HF placode [5]. The molecular nature of the earliest cues of HF-inducing signals from the dermis remains unclear [120]. Is it possible that the permanent part of the HF is also formed independently of E-M interactions? It is known that the formation of the epidermal placode from which the HF will be formed is specified by reaction-diffusion waves [121]. It is also well established that E-M interactions are indispensible for the formation of the temporary/cyclic component of HF in subsequent regeneration cycles. Therefore, it is tempting to postulate that the formation of the initial permanent component of the HFs is also independent of the E-M interactions.

Nascent velvet skin contains HFs [52], indicating that these are formed *de novo*. It would be interesting to know what chemical factors are involved in this induction. Interestingly, HFs that are formed in the velvet skin have much larger sebaceous glands, but there are no arrector pili muscles, and sweat glands [17, 122, 123]. This unique feature may help to decipher the origin of each component of the HF and offer some clues for the identification of the molecules that regulate the decision of stem cells to enter into different hair lineages and differentiation programmes for each lineage. In contrast to the process of HF morphogenesis, the cellular and molecular mechanisms that control the various morphogenetic events during early organogenesis of sebaceous glands are largely unknown [124].

Our studies showed that the E-M interactions in organogenesis and regeneration of AUs seem to be transient in nature because once antlers have transformed or regenerated from pedicles, physical separation of the two interactive tissue types does not stop antler generation [22] or regeneration [56]. How does this compare with HFs? Are the E-M interactions taking place in the organogenesis and regeneration of HFs also transient?

The close association between velvet epidermis and the PP in the AU does not immediately trigger antler regeneration, but rather the cycles enter a quiescent phase. This is because the endocrine factors (predominately androgens) override the outcome of the E-M interactions to suppress regeneration of antlers. Likewise, the close proximity of the DP and the bulge in HFs does not trigger regeneration of the temporal part of an HF, but the cycle enters a quiescent period called telogen, the length of which varies with species and follicle types. What factors suppress the outcome of the E-M interactions and determine the length of telogen for each HF type? If these overriding factors act in an endocrine or paracrine manner, then we must consider that different hair types may contain different receptors because HFs in different regions on a body have differing lengths of the telogen phase although they share the same systemic milieu. Factors involved in the BMP signalling pathway may also be implicated in this because there is increased activity in BMP signalling pathways to maintain HF stem cells in a quiescent state and these signals must be overcome to promote new tissue growth [116, 119]. Further research is required to clarify this hypothesis.

## Figures and Tables

**Table 1:** Overview of the comparisons between hair follicles and deer antlers.

Similarities		
Nature	Hair follicle	Antler unit
	Mammalian organ	Mammalian organ
Structure of mature organ	Permanent (infundibulum and isthmus) + cyclic (suprabulbar strand and bulb) components	Permanent (pedicle) + cyclic (antler) components
Ontogeny	Organogenesis and cyclic regeneration	Organogenesis and cyclic regeneration
Order of organogenesis	Permanent component formed first and cyclic component formed second	Permanent component formed first and cyclic component formed second
Nature of ontogeny	Stem-cell-based (bulge cells)	Stem-cell-based (AP and PP cells)
Location of stem cells	In permanent component	In permanent component
Initial identification of stem cells	Through tissue graft and genetic marker (LacZ gene) labeling, but not tissue deletion	Through tissue graft and genetic marker (LacZ gene) labeling, but not tissue deletion
Attributes of stem cells	Express embryonic stem-cell markers: Oct4, Nanog, and SOX2. Can differentiate into multiple cell lineages	Express embryonic stem-cell markers: Oct4, Nanog, and SOX2. Can differentiate into multiple cell lineages
Location of growth centre	In the cyclic component	In the cyclic component
Activation of generation and regeneration	By interactions between stem cells and the niche cell types (bulb cells)	By interactions between stem cells and the niche cell types (skin cells)
Process of appendage shedding	Enzymes are involved in a proteolytic process	Enzymes are involved in a proteolytic process
Endocrine control factors	Main factor: androgen	Main factor: androgen
Paracrine control factors	Main factor: IGF1	Main factor: IGF1
Molecules possibly involved in the interactions between stem cells and niche cells	Including canonical Wnt/*β*-catenin signaling, sonic hedgehog, and VEGF	Including canonical Wnt/*β*-catenin signaling, sonic hedgehog, and VEGF
Molecules possibly involved in maintenance of stem-cell stemness	Including c-myc	Including c-myc

Differences		
Nature of organ	Epithelium	Mesenchyme

Organ encapsulation	A layer of mesenchymal tissue	A layer of epithelial tissue
Order of structural components	Distal, permanent, proximal, cyclic	Distal, cyclic, proximal, permanent
